# Detection of Suicidality Among Opioid Users on Reddit: Machine Learning–Based Approach

**DOI:** 10.2196/15293

**Published:** 2020-11-27

**Authors:** Hannah Yao, Sina Rashidian, Xinyu Dong, Hongyi Duanmu, Richard N Rosenthal, Fusheng Wang

**Affiliations:** 1 Stony Brook University Stony Brook, NY United States; 2 Renaissance School of Medicine at Stony Brook University Stony Brook, NY United States

**Keywords:** opioid epidemic, opioid-related disorders, suicide, social media, machine learning, deep learning, natural language processing

## Abstract

**Background:**

In recent years, both suicide and overdose rates have been increasing. Many individuals who struggle with opioid use disorder are prone to suicidal ideation; this may often result in overdose. However, these fatal overdoses are difficult to classify as intentional or unintentional. Intentional overdose is difficult to detect, partially due to the lack of predictors and social stigmas that push individuals away from seeking help. These individuals may instead use web-based means to articulate their concerns.

**Objective:**

This study aimed to extract posts of suicidality among opioid users on Reddit using machine learning methods. The performance of the models is derivative of the data purity, and the results will help us to better understand the rationale of these users, providing new insights into individuals who are part of the opioid epidemic.

**Methods:**

Reddit posts between June 2017 and June 2018 were collected from *r/suicidewatch*, *r/depression*, a set of opioid-related subreddits, and a control subreddit set. We first classified suicidal versus nonsuicidal languages and then classified users with opioid usage versus those without opioid usage. Several traditional baselines and neural network (NN) text classifiers were trained using subreddit names as the labels and combinations of semantic inputs. We then attempted to extract out-of-sample data belonging to the intersection of suicide ideation and opioid abuse. Amazon Mechanical Turk was used to provide labels for the out-of-sample data.

**Results:**

Classification results were at least 90% across all models for at least one combination of input; the best classifier was convolutional neural network, which obtained an *F*_1_ score of 96.6%. When predicting out-of-sample data for posts containing both suicidal ideation and signs of opioid addiction, NN classifiers produced more false positives and traditional methods produced more false negatives, which is less desirable for predicting suicidal sentiments.

**Conclusions:**

Opioid abuse is linked to the risk of unintentional overdose and suicide risk. Social media platforms such as Reddit contain metadata that can aid machine learning and provide information at a personal level that cannot be obtained elsewhere. We demonstrate that it is possible to use NNs as a tool to predict an out-of-sample target with a model built from data sets labeled by characteristics we wish to distinguish in the out-of-sample target.

## Introduction

### Background

The ongoing opioid crisis is characterized by an increasing number of deaths caused by opioid overdose; this number increased to such an extent that in 2017, the US Department of Health and Human Services declared a public health emergency [1]. In 2016, the US Centers for Disease Control and Prevention reported 42,000 deaths caused by opioid overdose, resulting from both prescribed and nonprescribed opioids and including intentional (suicide) and unintentional (accidental) deaths [2]. Since 2008, suicide has been the tenth leading cause of death in the United States. In 2016, suicide became the second leading cause of death for those aged 10 to 34 years [3]. The American Foundation for Suicide Prevention approximates 44,965 Americans die by suicide each year, and for each suicide, 25 people attempt suicide. These numbers may be underestimated because of the stigma associated with suicide [4].

### Motivation

Individuals with chronic pain and poor mental health are often at risk of suicide; however, most current opioid overdose prevention methods neither assess suicide risk nor tailor prevention methods to personal situations [2,5,6]. Further hindering implementation of suicide screening is the lack of available data regarding death by overdose, frequently classified as *undetermined*, because of the inability to verify a death as a suicide. For example, if a deceased individual is found with a syringe by their side, it is difficult to determine whether it was an intentionally fatal dose or if the death was accidental (and without overdose intention) because of the risky route of administration by injection [6].

Individuals with opioid use disorder (OUD) are candidates for overdose due to the nature of the disorder. This study specifically focuses on suicidal ideation or intentions among individuals with a high possibility of OUD or, at minimum, opioid misuse. The Diagnostic and Statistical Manual of Mental Disorders defines OUD as a problematic pattern of opioid use leading to clinically significant impairment or distress [7]; approximately more than 2.5 million individuals are affected by OUD [8]. Diagnostic criteria include large amounts of opioids taken over a long period of time than intended, unsuccessful efforts to control opioid use, significant time investment in opioid-related activities or that necessary for recovery, impairment of daily life functionality, continued usage despite knowledge of consequences, dosage tolerance, and unavoidability of withdrawal [7].

The *New England Journal of Medicine* proposes that suicidal intention is blurred for individuals with OUD, who are repeatedly exposed to addiction-induced usage and withdrawal cycles and mood swings [2]. Ultimately, such prolonged exposure causes desensitization to risky and impulsive behaviors that can lead to death. Although the individual is aware of the consequences, the corresponding weight is muted and there is no associated conscious suicidal intent [9]. This aspect of OUD can lead to opioid overdose deaths but may not be explicitly considered in its contribution to overdose. Even in the absence of OUD, people who experience major depressive episodes, suicidal ideation, or other use and mental disorders are likely to misuse opioids, suggesting that opioid abuse and psychopathological conditions are self-perpetuating and cyclic behaviors [10].

Relevant predictors of suicide risk include chronic pain combined with depression [11,12]. Individuals with a history of depression also have a higher average daily dose and are more insistent on long-term opioid treatment for chronic pain [12], which is characteristic of OUD. Chronic pain and depression may also lead to suicidal thoughts even in originally healthy individuals [13], and both passive and active suicidal ideation are associated with mental illness, previous suicide attempts, and chronic pain [11,13]. Some suggest that opioid abuse manifests largely as a maladaptive coping strategy with physical and mental pain and is not solely biological [14,15]. Then, even if there is no conscious suicide intent, poor mental health leads the opioid-worn individual to adopt the mindset “I would not mind dying,” and they can become more reckless in their behavior, resulting in a higher probability of overdose. Given these correlations, it is not unreasonable to seriously consider suicide risk among opioid users.

Those with OUD who experience thwarted belongingness may not be willing to discuss their concerns with those in real life due to social stigma [16]. Instead, they may turn to web-based means of discussion, connecting with and seeking support from those who are more likely to understand them than health professionals [17]. Most opioid studies based on social media have been conducted on Twitter. However, Twitter is limited to 280 characters, which may not provide enough space for struggling individuals to express themselves thoroughly, and it can be highly noisy due to lack of monitoring and the medley of topics [18-22].

In contrast, Reddit is a forum-based social media platform for the discussion of many topics. Each topic has its own *subreddit* for topic-specific content, and the character limit for a text post is 40,000. Users can interact directly with an original post and with other users by commenting below the original submission. According to Reddit’s statistics page, it is currently the fifth most visited site in the United States, with more than 330 million monthly active users and 138,000 active communities [23]. Individuals are increasingly using mental health subreddits as a means of web-based support when offline support is unavailable. As Reddit also provides anonymity that is not available in real life, there is increased self-disclosure around stigmatic topics [24-26]. Furthermore, as subreddits are category-specific, struggling individuals can receive support and understanding from those with shared experience, who can provide therapeutic factors in the comments (eg, instillation of hope and altruism for those in opioid recovery or withdrawal) [27].

We consider the interpersonal psychological theory of suicide behavior as motivation for examining the connection between suicide and OUD [28]. In brief, this theory states that an individual will not die by suicide unless they have both the desire and the capability to act on that desire. According to the theory, suicidal desire is derived from an individual’s feeling of burdensomeness and an impeded sense of belongingness (social alienation) accompanied by the capability for suicide, which develops from repeated exposure to pain and exasperation [9]. Thwarted belongingness manifests as introspective torture in the individual with OUD, who often have ruined relationships or feels permanently labeled as *an addict* by society and cannot find understanding from those around them. The capability for suicide is acquired through active engagement with suicidal-like behavior and requires numbing out the innate sense of self-preservation [28]. This is applicable to those with OUD, many of whom have experienced overdose first hand or have witnessed their friends passing away from overdose and thus become desensitized over time.

### Objectives

To the best of our knowledge, the intersection of suicidality and OUD has not been analyzed in a data-driven fashion with machine learning methods, although both areas have been examined separately and research has been conducted clinically. We seek to use subreddits in combination with each other and machine learning methods to classify for in-sample data then predict for out-of-sample data to detect suicide ideation in the context of OUD.

In this study, we (1) utilized the structure and nature of subreddits to train classifiers with the goal of extracting posts containing both OUD and suicidal ideation, (2) compared inputs and models for classification, and (3) aimed to extract suicide risk posts from an opiate context and extract opioid addiction from a suicidal context, where *context* refers to the subreddit(s) to which the data set belongs. We also asked nonspecialist workers from Amazon Mechanical Turk (MTURK) to annotate a sample of posts to obtain labels for whether a post is indicative of both suicide ideation and opioid addiction.

### Related Work

Social media studies utilizing Reddit for its ability to provide massive amounts of text data have become popular. Many of these focus on current mental health concerns, such as depression and anxiety, and seek to analyze the linguistic features of post content and employ traditional methods such as N-grams, latent Dirichlet allocation topic modeling, linguistic inquiry and word count, term frequency-inverse document frequency (TF-IDF), and word embeddings to extract and analyze the emotional and mental states of Reddit users [25,27,29-31].

The subreddits r/suicidewatch and r/opiates revolve around the topics of suicidality (ie, discussions about, circumstance disclosure, emotional expressions, etc) and opioid usage (ie, drug doses, activities done while high, withdrawal anguish, etc), respectively. Analyses of specific subreddits have provided insights into the general mentality of subreddit users through their use of language. For example, a study by Park et al [29] showed that there are significant structure differences between sentences in mental health subreddits and nonmental health subreddits. They revealed differences between Reddit’s r/suicidewatch and the general mental health subreddit in their readability indexes and showed increased usage of first-person singular pronouns and decreased usage of first-person plural, second-person, and third-person pronouns [29]. Kumar et al [32] conducted a survey of r/suicidewatch to test for the Werther Effect, specifically following high-profile suicides, and found increased negative emotions, anger, self-harm, and suicidal words. They similarly reported increased usage of first-person singular pronouns along with decreased lexical diversity, longer posts, and fewer future-oriented words and more past-oriented words [32]. Yet another analysis of r/suicidewatch extracted recurring topics of discussion and then compared the results with suicide risk factors defined by domain experts; it was found that r/suicidewatch is capable of capturing less well-known dimensions of the risk factors, such as concerns about failing school, gun ownership, drug abuse, financial concerns, deceased friends, and domestic abuse [33]. The analysis of r/opiates yields dominant topics and characteristics of discussion, particularly for opioid users, such as opioid withdrawal and opioid intake routines [34].

These findings from r/suicidewatch support the findings of analyses of real-life writings left behind by people who attempted or completed suicide. In particular, a study of word use in poetry by suicidal and nonsuicidal poets revealed shifts in first-person singular pronoun and communication words (eg, *talk*), and a linguistic study of suicide completers showed that these individuals differ in time orientation compared to control groups [35,36]. The idea of *alienation* from the interpersonal theory of suicide supports the findings of increased usage of first-person pronouns—that when one is unable to relate to society, the presence of first-person plural *we* and other words of interaction decreases [28,37].

Neural networks (NNs) have been shown to achieve excellent results in natural language processing. Unlike traditional methods, *learning text from scratch* at the character or wording level requires no knowledge of sentiment; high-level targets can be used as input [38,39]. This is especially valuable in this context because drug-related words are often slang, and expressions related to getting high or intoxicated may be falsely categorized as negative when they are not due to the inability of sentiment lexicons to learn domain specificity [40]. Furthermore, there is no need for exhaustive feature engineering because weights can be learned [41].

Kim Yoon [42] demonstrated the ability of a simple convolutional neural network (CNN) with 1 layer of convolution in sentence classification for multiple data sets, finding that the performance of a simple CNN is comparable with that of traditional methods [42]. Orabi et al [43] used CNN and recurrent neural networks (RNNs) to predict depression for Twitter data, showing that CNN performs better than RNN. Johnson and Zhang [44] successfully used word sequences to classify documents with CNN, whereas Kim and Orabi only classified short, sentence length texts [44]. Singh et al [45] compared NNs and found that CNN performed best when considering both speed and accuracy. Therefore, we used a simple CNN architecture for its speed and performance.

## Methods

### Data Collection

The social media platform used in this study is Reddit. We focused on the subreddits r/suicidewatch and r/depression and opioid-related forums; also, a control group was used to model healthy, nondisordered language. The subreddit r/suicidewatch acts as a *bottom net*, such that if a user expresses suicidality, they will commonly be directed to r/suicidewatch for help. The subreddit has policies that forbid trolling and discourage activism (ie, repeatedly posting hotlines) in favor of direct peer support, which gives this forum credibility [46]. Subreddit r/depression is similar in forbidding *empty encouragement* (ie, *don’t worry, it gets better*). These characteristics exemplify the seriousness of these discussion boards and substantiate the reliability of the data. In contrast to these subreddits, however, r/jokes has few or no rules, only specifying that there are to be no personal attacks and stating that discussions must be lighthearted and civil; meanwhile, r/showerthoughts simply asks for original content. Posts in opioid-related forums were assumed to be indicative of individuals who struggle with opioid abuse and seek understanding on the web; our assumption is based on the fact that the act of posting about opioids on social media indicates that the presence of these drugs is sufficiently dominant in one’s life. All opioid-related forums were selected if the forum was relatively active and if the focus drug of the forum was frequently discussed in r/opiates, the most active opioid subreddit. Data were collected from Reddit using the pushshift.io Python Application Programming Interface (API) [47] and Reddit’s Python Reddit API Wrapper (PRAW) [48]. The IDs of submissions between June 2017 and June 2018 were obtained via pushshift.io and then passed to PRAW, which retrieved the actual submission. A submission consists of an ID, author, title, and body text/content.

We used posts from r/suicidewatch to represent suicidal language and a collection of opioid-related subreddits to model the language of opioid users (which is referred to in this study as r/opiates or *opiates data*). We assumed that all posts from r/suicidewatch were suicidal and that the language of r/depression would be most similar to that of r/suicidewatch [49]. Several control subreddits were used for language comparison based on a study by Shen et al [50] for detecting anxiety on Reddit, which implies that subreddits themselves can be used as labels. This set of diverse subreddits seeks to offset the impact of excessive first-person pronoun usage that is common when expressing negative emotions and subject-specific words [51,52]. We assumed that the majority of posts in these control subreddits were *mentally healthy*, meaning that post content (saying nothing about the user themselves) is not reflective of mental disturbances such as depression and suicidal ideation. In addition to the selected subreddits from those used by Shen et al [50], we included r/careerguidance and r/personalfinance to account for finance stressors that may be experienced by those in r/suicidewatch, and we included r/offmychest to account for the potentially rougher and more profane language of opioid users, which is indicative of strong negative emotions but unlikely to indicate suicidality. Examples of posts in some specific subreddits are shown in Table 1, and the data groups and their subreddits are listed as follows:

- *Depression*: depression.
- *Suicidewatch*: suicidewatch.
- *Control*: askdocs, askscience, books, careerguidance, fitness, frugal, jokes, lifeprotips, offmychest, parenting, personalfinance, productivity, randomkindess, relationships, showerthoughts, talesfrom retail, theoryofreddit, wholesometextposts, writing, youshouldknow.
- *Opiates*: benzodiazepines, benzorecovery, heroin, methadone, opiates, opiatesrecovery, quittingkratom, suboxone.

**Table 1:** Example posts from subreddits belonging to data groups.

Subreddit group	Title	Body text
Control (r/showerthoughts)	It becomes less and less acceptable to cry in public the older you get, despite the reasons for doing so becoming more and more valid	Kids just don’t understand
Opioid related (r/opiates)	Happiness is…a big, dark shot of Heroin after being sick all week. <3	And also knowing you have enough for not only the next morning but at least a few more days while you figure out what to do next!
Depression	I won’t commit suicide but I wouldn’t mind dying	So much shit has been piling on and on. I feel like I am not making the people I care about proud and the only reason they talk to me is because of pity. I will not take my own life, but if a car hit me, I got terminal illness or if something fell on me. I would not be sad about me being gone
SuicideWatch	I’m waiting for the courage to end my own life	I feel like I’m close to making a serious attempt sooner than later and I’m ok with that. My impulsive behaviors have gotten worse other the last few months and some of those ways include bodily harm. In September i impulsively jumped out of a friends window and injured myself and now I am cutting myself at random for the first time in years. No one around me understands how exhausting it is to wake up everyday in my own skin. In my own head. I’m sick of the stomach pangs and guilt and crying and disappointment. Some nights I just pray I’ll have the courage to end it. People die in the world all over, shouldn’t matter when I go

### Feature Matrix Construction

We maintained the high-level structure of the language, only lowercasing and removing URLs [42]. The text was not lemmatized, and all stop words and profanity were kept. All punctuation marks were removed except for periods, commas, exclamation marks, question marks, and apostrophes. We sought to make use of word order for text categorization [44]. As the title of a post may be a good representation of post content, the title of every post was prepended to the body text. The joined title and body content composed the NN input. All posts were either zero padded or truncated to a length of 1500 words, as we wanted to use the entire post if possible. Each post (title+body text) was preprocessed, tokenized, and encoded, preserving the word sequence, and is thus represented as a vector of words. We also reran the classification using only posts with more than 1000 words to show that differing content lengths do not impede performance. Textbox 1 shows examples at each text-processing stage.

**Example of text-processing stages.**

Unprocessed text

- Question. What’s the best otc med to OD on? I’m so over the day to day grind. I’m a failure at life and ready to check out...

Processed text

- Question. what’s the best otc med to od on ? i’m so over the day to day grind . i’m a failure at life and ready to check out . . .

Tokenized

- | question | . | what’s | the | best | otc | med | to | od | on | ? | i’m | so | over | the | day | to | day | grind | . | i’m | a | failure | at | life | and | ready | to | check | out | . | . | . |

As sequential text data cannot be used as is for training machine learning models, there are several techniques that can convert these data to numerical values:

1. TF-IDF: The term frequency-inverse document frequency shows how important a word is in a document with respect to its frequency in other documents. We used a 100-dimension TF-IDF from in-sample unigrams and bigrams [53].
2. Word embedding: This is a set of methods and language models for converting text data to vector space. We took advantage of 3 methods, including the Gensim Word2Vec model Global Vectors for Word Representations (GloVe), which require embeddings to be trained in advance on a large corpora of data and also a simpler version of word to ID mapping, which was performed in running time [54,55].
3. Character embedding: As out-of-dictionary words are all mapped to random vectors in pretrained word embeddings or the slightest change in slang language could end up mapping words to different vectors, adding character embedding as well showed improvements for training the machine learning models.

In this study, we took advantage of different combinations of these knowledge representation methods to evaluate whether classification performance improves additional information by alleviating vocabulary issues. Multimedia Appendix 1 shows the complete list of combinations.

#### Models

Our main focus was on a CNN text classifier. In addition, we implemented several traditional and deep learning models to serve as baselines for the CNN. Details are as follows.

##### Classic Baselines

We employed well-known traditional machine learning methods as baselines, including logistic regression (LR), random forest (RF), and support vector machines (SVMs). The implementation used Python language and was based on the scikit-learn toolkit [53].

##### NN Baselines

We implemented FastText (FAST), RNN, and attention-based bidirectional RNN (ATTENTION) in their original architectures for comparison [56-58]. FAST uses a shallow NN and word representations constructed from bag-of-n-grams, which are averaged into the text representation and input to a linear classifier. RNN considers historical information and shares weights across time by allowing previous outputs to be reused as inputs. ATTENTION builds on RNN by introducing the ability to *attend* to specific subinputs rather than all available information, thereby improving the decisions made at each step.

##### CNN Model

**Figure 1:**
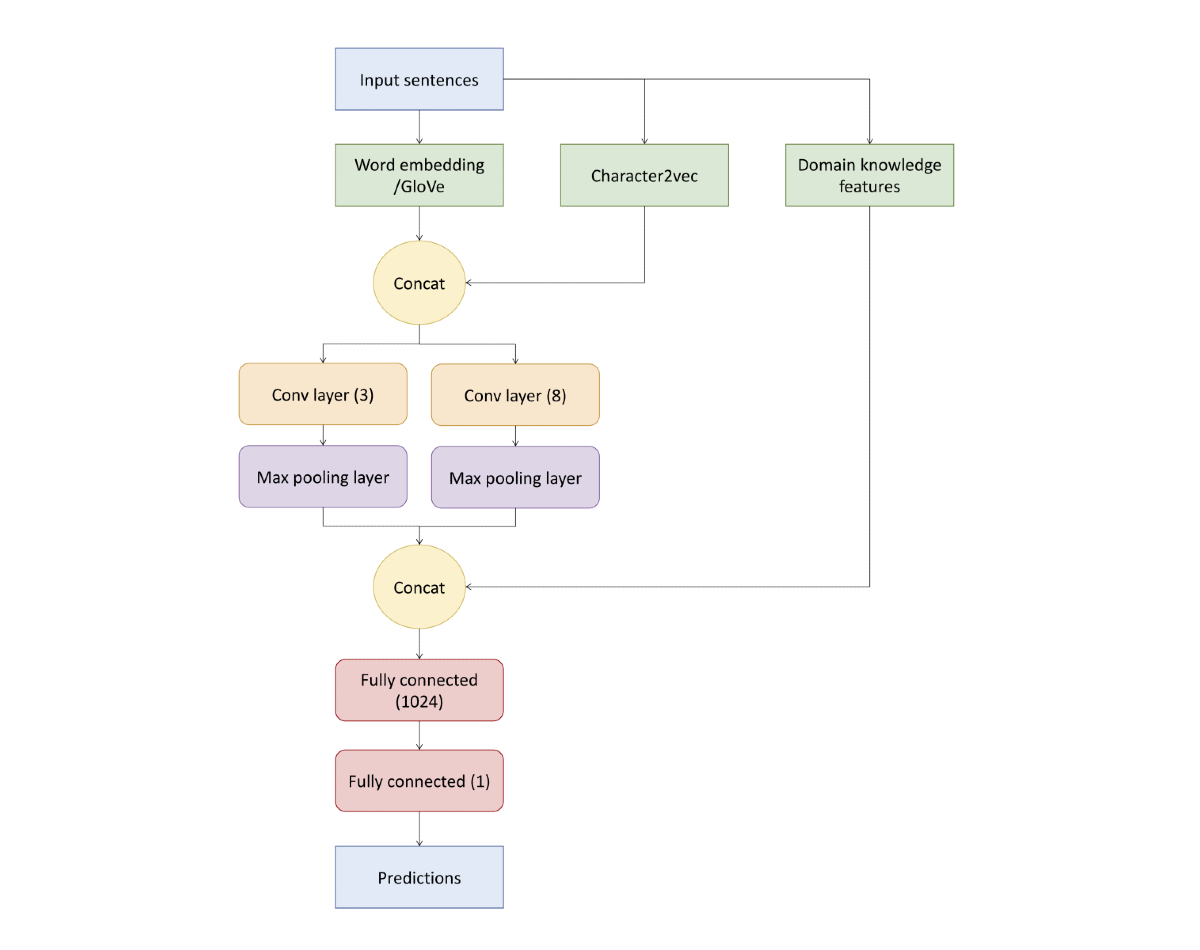
Overview of the convolutional neural network architecture.

We implemented a CNN based on Kim Yoon’s CNN architecture and used the Keras NN library in Python [59]. The major difference between this implementation and Kim’s is that random initialization was used instead of word2vec because both perform equally, max pooling was used instead of global pooling, and filter sizes of 3 and 8 were used instead of 3, 4, and 5. A filter size of 8 was shown to work better with longer documents and would slide over a window size of 8 words at a time [60]. The activation layer following every convolutional layer was the rectified linear unit, f(x)=max(0,x), which maps nonnegatives between the input and feature map. In terms of integrating additional information, domain knowledge features and character2vec (char2vec), into the original CNN system, we simply used concatenation. As the char2vec feature matrix has the same size as the word embedding features and similar logical meaning, we concatenated them together before the main convolution part. Domain knowledge features, a large vector showing the information extracted from the linguistics perspective, were concatenated before the final fully connected layers part. We used a batch size of 512, epoch count of 6, and embedding layer that was learned at the training time. The embedding layer had a dropout of 0.5 to prevent overfitting and a dimension size of 100. Loss was calculated using cross-entropy. Figure 1 shows our CNN architecture.

#### Classification and Prediction

Our hypothesis is from an intuitive standpoint. We considered 2 cases: (1) there exist posts with suicide sentiments among the opiates data set and (2) there exist posts indicative of opioid addiction among the suicidewatch data set. We sought to extract posts that contain both suicidal sentiments and opioid abuse. These 2 cases are referred to as *C1* and *C2* and are represented by their respective models.

Case 1 (C1) is a model that will be trained on data from r/suicidewatch and the control subreddits for positive language, and it will learn to distinguish between suicidal and positive language. As r/opiates contain mixed sentiments, if we feed the out-of-sample data from r/opiates into C1, then C1 should be able to classify between suicide risk posts and nonsuicide risk posts. Posts from r/opiates that are classified as *suicidal* will then contain both suicidality and OUD aspects. In addition, we coarsely assessed the quality of posts predicted as *suicidal* by constructing a word2vec from predictions.

Case 2 (C2) is a model that is trained on data from r/depression and r/opiates. The subreddit r/depression is the most similar to r/suicidewatch which is available. C2 will learn to distinguish between posts containing drug addiction and no drug addiction. If we feed out-of-sample data from r/suicidewatch into C2, then C2 should be able to classify between posts containing opioid usage and nonopioid usage. Posts from r/suicidewatch that are classified as having opioid usage will then contain both suicidality and OUD aspects.

For classification, the data sets were split with an 8:2 ratio for training and testing. The data for training were split again with an 8:2 ratio for train versus train validation. To evaluate the prediction ability, we sampled 500 posts ad hoc from r/opiates and 500 posts from r/suicidewatch to be annotated. To enforce a lower bound on sample quality and reduce variance among the posts, we only sampled from posts between 30 and 500 words. As class balance is a concern within a sample of 500, a maximum of 250 of 500 posts were selected if they contained a keyword or a key phrase of interest. Thus, the 8:2 train/test split over the entire data set was used for in-sample testing, and then 500 samples were extracted from the 20% of posts that belong to the test split for out-of-sample testing. Table 2 summarizes the C1 and C2 procedures and is illustrated in Multimedia Appendix 2, which demonstrates the workflow for C1.

**Table 2:** Summary of the 2 models for the 2 cases.

Case		C1	C2
**Training and classification**			
	Goal	Distinguish between suicidal and nonsuicidal language; predict for suicidality among opioid users	Distinguish between language of opioid usage and depressed but nonopioid using; predict for opioid usage among suicidal individuals
	Data set	51,366 posts from r/suicidewatch and control subreddits	59,940 posts from opioid relevant subreddits and r/depression
Vocabulary size		70,082	64,078
Training		Trained and validated on data from r/suicidewatch and control subreddits	Trained and validated on data from opioid relevant subreddits and r/depression
**Prediction on out-of-sample data**			
	Predicts on	Data from r/opiates	Data from r/suicidewatch
	Prediction goal	Predicts for posts containing suicide risk in r/opiates	Predicts for posts containing opioid abuse in r/suicidewatch
	Total posts prediction data that are between 30 and 500 words	23,740 posts from r/opiates	21,719 posts from r/suicidewatch
	Sample max 250 from data for prediction containing these keywords for MTURK^a^	Commit suicide, suicide, suicidality, suicidal, want to die, want to do, want to overdose	Benzodiazepines, benzos, cocaine, codeine, fentanyl, heroin, hydrocodine, hydrocodone, hydromorphone, hydros, kratom, methadone, morphine, narcotic, narcotics, opiates, opioid, oxycodone, oxycontin, oxycottin, oxycotton, oxymorphone, suboxone
Sample count containing keywords		234	231

^a^MTURK: Amazon Mechanical Turk.

### Annotations With Amazon Mechanical Turk

The sampled posts were annotated by crowdsourced workers from MTURK [61]. Each post was annotated by 3 workers, and the final label for a post was determined by the majority rule.

For C1, the MTURK task was titled “Suicidal sentiment detection.” The description was “May the user be at risk of suicide or intentional overdose?” with 2 options: “Yes, risk of suicide” and “No risk of suicide.” Task keywords included *depression, drug abuse, drugs, mental, mental health, opiates, overdose, suicide, suicide ideation, suicidewatch,* and text. Eligible MTURK workers were required to have masters qualifications and were awarded US $0.04 per annotation. We required no other qualifications. Assuming that the average individual lacks experience in both mental health and drug misuse [62], we defined common slang terms from r/opiates (eg, PAWS stands for postacute withdrawal syndrome) and provided simple instructions based on *Clues to Suicide* by Edwin Shneidman, who is the founder of the American Association of Suicidology and has laid groundwork in the field [63].

For C2, the MTURK task was titled “Opioid addiction among suicidal individuals.” The description was “Does the post imply opioid addiction?” with 2 options: “Yes, implies opioid addiction” and “No opioid addiction.” Task keywords and qualifications were the same as in the C1 MTURK tasks. We provided a list of opioid names in the instructions.

## Results

### Comparison of Models and the Impact of Feature Matrices

Metrics were calculated for accuracy, precision, recall, *F*_1_ score, and area under the curve. The complete results can be found in Multimedia Appendix 1. Table 3 provides a subset of the results of comparing the CNN with the traditional baselines using the *F*_1_ score in training the classifiers. Table 4 provides the results of comparing the NNs. At the end of this phrase, the models had been trained and were ready to be used for prediction of out-of-sample data.

**Table 3:** *F_1_* scores achieved for the given different combinations of input for classification.

Model		LR^a^	RF^b^	SVM^c^	CNN^d^
**C1: r/suicidewatch versus positive control group**					
	TF-IDF^e^	0.902	0.904	0.915	0.685
	word2vec	0.928	*0.927* ^f^	*0.943*	0.961
	TF-IDF + word2vec	*0.940*	0.921	0.941	*0.963*
	TF-IDF + GloVe	0.927	0.829	0.886	0.923
	TF-IDF + word2vec + char2vec	0.914	0.790	0.856	0.962
**C2: r/depression versus r/opiates**					
	TF-IDF	0.889	0.800	0.811	0.729
	word2vec	0.852	*0.846*	*0.881*	0.961
	TF-IDF + word2vec	*0.894*	0.815	0.880	0.965
	TF-IDF + GloVe	0.860	0.494	0.765	0.814
	TF-IDF + word2vec + char2vec	0.858	0.581	0.741	*0.966*

^a^LR: logistic regression.

^b^RF: random forest.

^c^SVM: support vector machine.

^d^CNN: convolutional neural network.

^e^TF-IDF: term frequency-inverse document frequency.

^f^The best results achieved by the model are in italics.

**Table 4:** Comparison of text classification neural network baselines with word embedding and convolutional neural network.

Model		FAST^a^	RNN^b^	ATTENTION^c^	CNN^d^
**C1: r/suicidewatch versus positive control group**					
	Accuracy	0.950	0.944	0.939	*0.954* ^e^
	Precision	0.958	0.953	0.934	*0.968*
	Recall	0.957	0.951	*0.965*	0.953
	*F*_1_ score	0.957	0.952	0.949	*0.961*
**C2: r/depression versus r/opiates**					
	Accuracy	0.971	0.957	0.969	0.971
	Precision	0.964	0.964	0.967	0.970
	Recall	0.958	0.923	0.951	*0.962*
	*F*_1_ score	0.961	0.943	0.959	*0.966*

^a^FAST: FastText.

^b^RNN: recurrent neural network.

^c^ATTENTION: attention-based bidirectional recurrent neural network.

^d^CNN: convolutional neural network.

^e^The best score for each model is italicized.

From Table 3, we can observe that word2vec contributed to the CNN performance. Another interesting observation is that the classification performance of all models was affected when a pretrained GloVe was used as input. This is possibly due to the local information introduced by using specific subreddits as categories. The introduction of GloVe and character embeddings impeded the performance of RF, which implies that RF experiences more difficulty in learning implicit semantic features than LR and SVM. In contrast, NNs performed well with semantic-based inputs.

**Figure 2:**
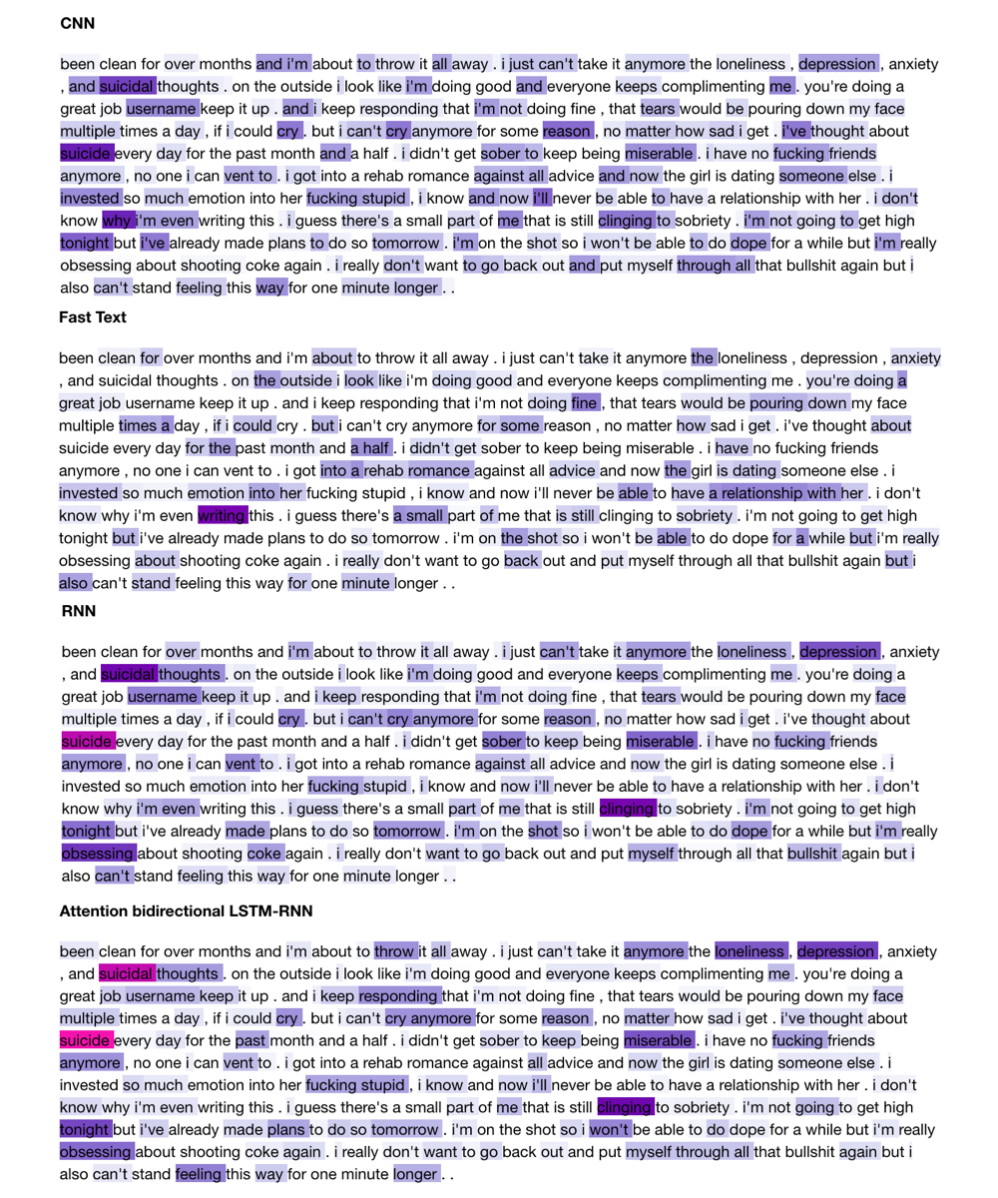
Visualization of word importance as determined from the resultant word embeddings learned from scratch by the neural network models for classification of suicidal vs non-suicidal text. CNN: convolutional neural network; LSTN: long short-term memory; RNN: recurrent neural network.

Figure 2 provides a visualization of the word embeddings that the NNs trained from scratch. Color intensity and shade from light purple to hot pink indicate increasing emphasis placed on the respective words. CNN, RNN, and ATTENTION share many important words, whereas FastText appears to focus on different words given its bag-of-N-grams approach but nonetheless achieved high classification performance.

### Evaluating Predictions Using MTURK Annotations

For prediction, we used traditional baselines with word2vec input and NNs. To assess the prediction performance, MTURK was used to obtain labels with 3 annotations per post. C1 predicted for suicidality among opioid data. Annotations were performed by 25 unique workers, with an average of 4 minutes spent on each post. The pairwise agreement among the 3 annotations was 0.632, 0.628, and 0.644. In total, 120 out of 500 posts were ruled as yes, suicide risk. C2 predicted opioid addiction among suicidal posts. Annotations were performed by 92 unique workers, with an average of 11.3 minutes (C1: SD 6.667 minutes; C2: SD 14.633 minutes) spent on each post. The pairwise agreement among annotations was 0.524, 0.604, and 0.544. In total, 261 out of 500 posts were ruled as *yes, implies opioid addiction*. Table 5 shows the model performance using the majority rule MTURK annotations as the ground truth. Table 5 also shows the differences in performance depending on the ratio of labels in the data set. To construct a set of data with 500 rows with a certain percentage of suicide risk posts, we sampled using sckit-learn with replace as true.

**Table 5:** Count and accuracy for model predictions using Amazon Mechanical Turk labels.

Model		LR^a^	RF^b^	SVM^c^	FAST^d^	RNN^e^	ATTENTION^f^	CNN^g^
**Count and accuracy for C1 (predicts for suicide risk among opiates data)**								
	Predicted number of suicide risk	24	12	11	97	93	98	103
	All data	0.768	0.750	0.744	0.59	0.608	0.576	0.536
	Suicide risk only	0.2	0.1	0.092	0.783	0.791	0.75	0.833
	Nonsuicide risk only	0.947	0.959	0.963	0.529	0.55	0.521	0.432
**Count and accuracy for C2 (predicts for opioid addiction among suicidewatch data)**								
	Predicted number of opioid addiction	88	92	105	92	158	110	127
	All data	0.524	0.518	0.538	0.54	0.588	0.552	0.562
	Opioid addiction	0.230	0.237	0.273	0.251	0.414	0.295	0.334
	Nonopioid addiction	0.892	0.869	0.869	0.9	0.806	0.874	0.847

^a^LR: logistic regression.

^b^RF: random forest.

^c^SVM: support vector machine.

^d^FAST: FastText.

^e^RNN: recurrent neural network.

^f^ATTENTION: attention-based bidirectional recurrent neural network.

^g^CNN: convolutional neural network.

### Observations Regarding C1: Detecting Suicide Ideation in the Context of Opioid Addiction

For both C1 and C2, we noticed that the model metrics changed drastically when the ratio of posts per label was altered. For C1, traditional baselines performed better when there was a mixture of suicidal and nonsuicidal-labeled posts because NNs produce a high number of false positives. However, when only examining suicidal posts, NNs achieved better accuracy. Thus, depending on the domain and data set, it may be better to use models that are superior at the extremes or remain within a relatively less variable range. For our case of predicting suicide risk posts among the out-of-sample opiate data, it is better to err on the safe side than to miss someone who is at risk of suicide. CNN would be a good choice if the sample consisted of many suicide risk posts. The predictive power of the baselines would not be desirable when predicting for posts indicative of suicide risk, as false negatives would be costly in the context of real life.

Given that the models tended to predict at the extremes for C1, we speculated that the models would serve well as weak learners to an ensemble model. We explored a simple ensemble model using scikit-learn’s AdaBoost Classifier and LR, SVM, RNN, and CNN as weak learners. We used MTURK-labeled data of size 500 as the ground truth and the weak learner’s probabilistic predictions as the input data. The data were split into training and testing sets at an 8:2 ratio. AdaBoost was run 5 times with different data shuffles and random states, and the average accuracy was taken.

**Figure 3:**
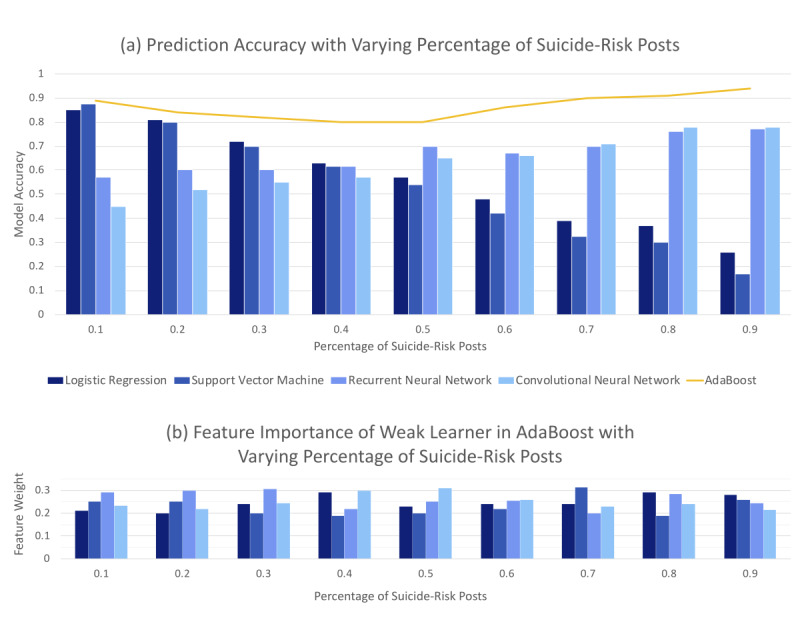
The top bar graph shows changes in prediction performance in C1 depending on data category ratios. The overlaying line plot shows accuracy achieved from using the four models as weak learners in AdaBoost. The bottom graph shows weak learner contribution.

In Figure 3, the top graph illustrates the performance of selected models as we increased the percentage of suicide risk labels from opiate data in C1 from 10% to 90%. The line plot shows the performance of AdaBoost with those models as weak learners. AdaBoost achieved respective accuracies of 0.89, 0.84, 0.82, 0.8, 0.8, 0.86, 0.9, 0.91, and 0.94 for the respective intervals. The bottom graph in Figure 3 displays the model contribution at each step. From the graph, combining the models that predict at the extremes results in a more robust classifier. This is an advantageous approach because the ensemble model now has knowledge about the out-of-sample data, which the weak learner models did not have.

**Figure 4:**
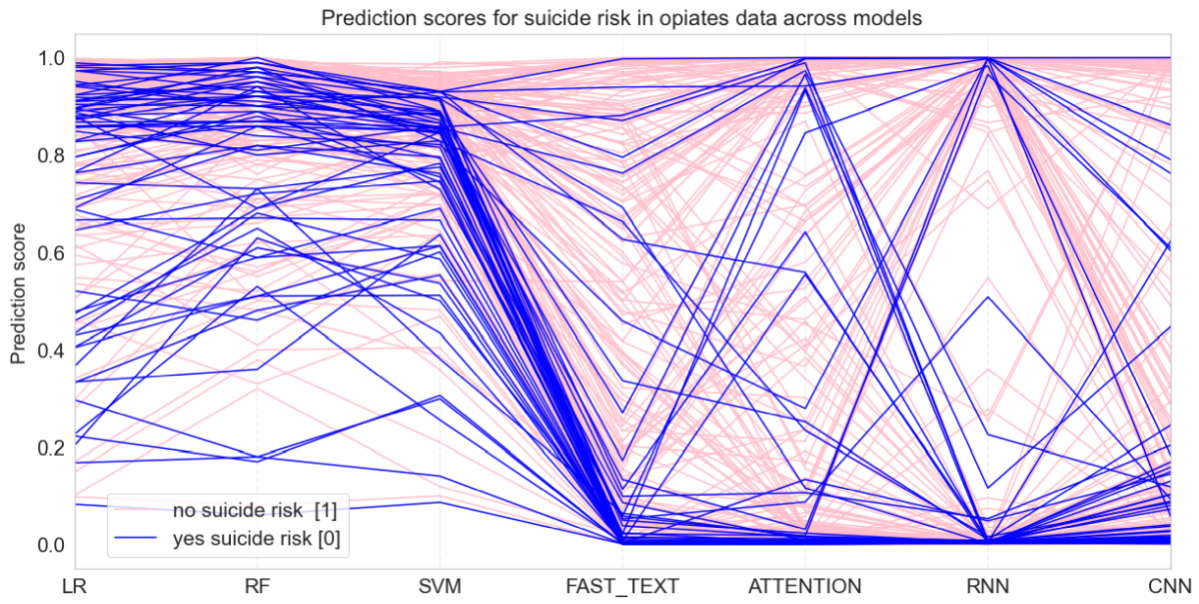
Model predictions for individual posts represented as a line. A prediction closer to 1 indicates no predicted suicidal language and a prediction closer to 0 indicates yes predicted suicidal language. Individual posts are color coded by their MTURK label. ATTENTION: attention-based bidirectional recurrent neural network; CNN: convolutional neural network; FAST_TEXT: FastText; LR: logistic regression: RF: random forest; RNN: recurrent neural network; SVM: support vector machine.

Figure 4 shows the parallel prediction performance for C1 at a more fine-grain level; 50% of each label was sampled for the visual. Each line represents a post. The categorical *x*-axis lists the models, and the intersection of a line with the *y*-axis of a model is the prediction score of the model. The darker lines are MTURK-labeled as indicative of suicide risk (0 on the y-axis), whereas the lighter lines are nonindicative of suicide risk (1 on the y-axis).

### Observations Regarding C2: Detecting Opioid Abuse in the Context of Suicide Ideation

Our hypothesis for C2 was that after being trained on data from r/depression versus the opiates group, the models would be capable of detecting phrases used by individuals who struggle with drug addiction. Although the models all achieved good results in classifying r/depression versus opiate data, they failed to extract opioid-containing posts with respect to the MTURK labels. One possible explanation is that users express themselves differently in different contexts (subreddits). Another possibility is that the language semantics in r/suicidewatch forgo characteristics found in opiates, such as details about withdrawal or relapse that take up the majority of space in any opiate context post. For example, a post in r/suicidewatch may briefly mention a few keywords such as “hydros” or “benzos” in a sentence about overdosing, while the remainder of the post focuses on personal struggles. On the other hand, if a user from r/opiates posts about wanting to commit suicide in r/opiatesrecovery, they will express sentiments regarding the drug because they know their audience will understand. It is possible that the user is subconsciously assuming that the audience in r/suicidewatch knows little about opioid addiction and therefore omits the consequences of opioid addiction on their life. As a result, an MTURK worker who reads the entire post may observe that the user has easy access to opioids and is experienced with drug mixing; however, the model may fail to pick up such meanings because these exact expressions did not appear in the data it was trained on.

From our new hypothesis regarding language usage in C2, we re-evaluated the C2 predictions. Instead of using MTURK-determined labels, we constructed a set of heuristic labels such that a post is implicative of opioid addiction if the post contains any word from the C2 keywords in Table 2 and is not implicative of opioid addiction otherwise. The findings are shown in Table 6. Although the overall performance was not ideal, all scores increased, supporting our hypothesis that the presence of specific words is important and that MTURK workers’ directed attention likely differs from that of the models.

**Table 6:** Model prediction accuracies with heuristic labels determined by the keyword presence.

Model	LR^a^	RF^b^	SVM^c^	FAST^d^	RNN^e^	ATTENTION^f^	CNN^g^
All data	0.606	0.588	0.648	0.666	0.774	0.69	0.712
Opioid addiction	0.264	0.26	0.347	0.338	0.56	0.403	0.463
Nonopioid addiction	0.9	0.87	0.907	0.948	0.926	0.937	0.926

^a^LR: logistic regression.

^b^RF: random forest.

^c^SVM: support vector machine.

^d^FAST: FastText.

^e^RNN: recurrent neural network.

^f^ATTENTION: attention-based bidirectional recurrent neural network.

^g^CNN: convolutional neural network.

### Content Overview for Predicted Suicidal Posts in Opiate Data

We constructed a bigram word2vec from posts predicted as *suicidal* in the entire opiate data set (opiate data: n=23,740, as shown in Table 3) to holistically assess the quality of these posts. We then queried for the top 15 most similar words to “suicidal” for each model, as it is more likely to appear with first-person pronouns as opposed to other derivations of the root “suicid.” Table 7 summarizes the results from the most similar to least similar words.

**Table 7:** Top words most similar to suicidal from the subset of opiates data that was predicted to belong to the category suicidal.

Model	Top words
LR^a^	hour, dead, lack, asleep, wake up, sobriety, self, they are, cause, at least, however, hell, later, group, state
RF^b^	easy, living, yet, hit, waiting, probably, cold, the same, such, by, tomorrow, body, constantly, saying, working
SVM^c^	making, everyone, once, pills, without, soon, lol, nothing, around, sorry, thing, withdrawal, start, mental, tolerance
FAST^d^	suicidal thoughts, depressive, extreme, emotional, severe anxiety, existing, depressed, irritable, insomnia, severe, nauseous, mood swings, paranoid, fatigued, having trouble
RNN^e^	severely, depressed, diagnosed with, isolated, unbearable, bipolar, suicidal thoughts, anxious, ptsd, an alcoholic, ocd, overwhelmed, irritable, lethargic, extreme
ATTENTION^f^	diagnosed with, depressive, suicidal thoughts, social anxiety, bipolar, extreme, crippling, irritable, tremors, emotional, borderline, severe depression, brain fog, existing, gad
CNN^g^	paranoid, unhappy, depressed, isolated, apathetic, irritable, an alcoholic, suicidal thoughts, trauma, severely, diagnosed with, brain fog, anxious, manic, emotionless

^a^LR: logistic regression.

^b^RF: random forest.

^c^SVM: support vector machine.

^d^FAST: FastText.

^e^RNN: recurrent neural network.

^f^ATTENTION: attention-based bidirectional recurrent neural network.

^g^CNN: convolutional neural network.

## Discussion

### Overview

The goal of this study is to make use of the structure and data from Reddit to build models that could ultimately predict (C1) suicidal language among opioid users and (C2) opioid usage among individuals with suicidal thinking. We evaluated several models with combinations of semantic inputs and well-known NN text classifiers in their ability to classify and then predict out-of-sample data to extract posts containing both suicide ideation and opioid abuse. MTURK was then employed to provide heuristic ground truths to the out-of-sample data.

### Limitations

Owing to the large quantity of data, we assumed that all posts under a subreddit were reflective of the official subreddit purpose (eg, recovering opioid users post in r/opiatesrecovery) and that deviations were neutralized because of the sheer volume of the majority content. A manual review of predicted suicidality among opioid subreddits (C1) found that many cases were wrongly predicted as suicidal because the users were experiencing extreme withdrawal, anger due to relapse, and even anger due to sobriety because of the inability to use opioids as a coping mechanism. Inclusion of r/offmychest in the control subreddits was an attempt to offset such strong negative emotions that likely do not imply suicidality. A suicidal post may also be predicted as nonsuicidal because of calm resignation language or because the user is writing in self-reflection or to share past experiences.

A manual review of predicted opioid usage in r/suicidewatch (C2) found that poor predictions may have resulted from the sentiment mixture present in the opioid reddits. As the opioid group comprises active users and users in recovery/withdrawal (ie, r/opiates vs r/opiatesrecovery), the language variation could be extreme and vary from anger (ie, My dealer is ****ing late) to negative (ie, *I can’t find happiness outside of heroin*) to joy (ie, *I’ve been clean for 70 days!*). All of these posts were given the same label of *opiates* as they were grouped under opioid language. Hence, the presence of drug-specific words is needed to predict opioid usage.

#### MTURK Limitations

We attempted to overcome MTURK limitations by providing brief context and instructions. Regardless, limitations included lack of domain knowledge, experience with suicide ideation or drug misuse, and subjectivity of interpretation. There is also possible carelessness in annotation, as some posts were relatively long and not all posts may have been read thoroughly. Furthermore, we provided a single post out of context from the user’s life; it is difficult to determine how alarming the post is in reality because we cannot know what happened to the user later in time. There was also no way to control how many and which users participated in the assessment—the participation of many distinct workers may have introduced random variations in annotation consistency. Manual review of several posts that were disagreed upon showed that lack of domain expertise can make annotation difficult; for example, a post that was disagreed upon mentioned being *tired of waking up*, in *withdrawal*, and that it is *easier to end it all*, but also mentioned *wanting to get sober*. One MTURK worker may have considered this post as hopeful, whereas another worker may have considered the post to be on the edge of giving up. Despite these limitations, these annotations provide insight into the public perception regarding what is considered suicidal when opiate addiction is involved.

#### Insights

Studies conducted on opiates and other drug-addicted individuals have shown that opiate addiction is often comorbid with other psychological disturbances, such as personality disorders or trauma [64,65]. We observed this in the simple overview of words most related to *suicidal.* Among the NNs, several personality disorder terms and mental illnesses were extracted, such as *ptsd* (posttraumatic stress disorder), *borderline, bipolar*, and *gad* (general anxiety disorder). Characteristics of withdrawal were also captured: *irritable, insomnia, nauseous*. We can speculate that individuals with personality disorders going into withdrawal phrases are at higher risk of attempting suicide than other subgroups of the population.

### Future Directions

There are many possible directions for utilizing Reddit metadata. For subreddits with mixed emotional sentiments such as r/opiates, coarse grain classification can first be applied to discard either too positive or negative posts depending on context. Given the interactivity of Reddit, analyses of comments may show what sort of support is provided by peers who struggle. The peer aspect of Reddit is attractive, and integration of nonjudgmental peer support may help recover OUD users to be more willing to rely on real-life aid. This is one step toward decreasing the frequent social isolation brought upon by OUD.

In general, exploration of Reddit data limits provides many possible study areas, given the amount of active subreddits. Among all posts in r/opiates, we found a handful in which a user who has successfully quit opioid use for many years and returns to relay hope for a full recovery. Such individuals can be seen as *success stories.* It is of particular interest to examine the difference between these individuals and those who end in tragedy, as OUD recovery is difficult and relapse prone [66]. One could also consider the interaction among suicide ideation, OUD, and chronic pain, especially because many OUD cases begin with an individual’s or close person’s prescription medication [10]. Another focus is the possibility of influencing the OUD individual’s behavior; a study in the context of behaviorally targeted advertising for health care based on user searches implies that behavior, as determined by input search terms, is predictive of the individual being influenced by the target advertisement to act, such as visiting a health provider [67]. Therefore, internet behavior may be more closely linked to real-life behavior than expected, and targeted advertising might encourage OUD individuals who are midwithdrawal and fighting relapse to seek help.

The tendency of NNs to achieve high accuracy among purely suicide-labeled posts but perform poorly when the data set is mixed prompts the question of which semantic dimensions are actually being captured. Although we use NNs as a black box in this experiment, future studies may want to focus directly on latent semantic dimensions that may aid in parameter tuning and generalizability. In particular, it would be very interesting to extract the exact features that might generalize across textual data domains by examining the word embeddings that the NNs had trained from scratch to aid in predictions on out-of-sample data [68].

We focused on extracting posts that are indicative of suicide ideation in the context of OUD and did not perform much analysis on extracted posts. Many studies have investigated the content of suicidal individuals, but much fewer studies have been conducted in specific contexts, possibly due to the lack of available data. Gathered predictions are capable of revealing thoughts of suicidal opioid users, despite the limitations of this study. In aiding those recovering from OUD, it may be important to consider beginning at the personal level [69]. Communication between the professional and the patient is important, and personal stories are capable of decreasing stigma by deconstructing stigmatic barriers [70]. As these posts are likely to be raw and honest, they can allow clinical professionals to become more familiar with the mindsets of these individuals. A less stigmatic perception of patients can lead to patient empowerment, which is necessary in the long term [66,71]. Consider a post that was agreed upon as showing the risk of suicide, summarized quote:

as soon as something goes missing, or something goes wrong, you're the first fucking person everyone suspects is at fault…it MUST have been the fucking drug addict, right? … I may be a dopehead, but I have never been a thief.

It is easy to advocate understanding and help for those struggling with opioid addiction; however, it is harder to recognize how their own actions may affect those with opioid addiction. We believe that concrete case examples are essential as a first step and that suicide risk assessment should be done on an individual level. Our study seeks to aid in suicide prevention by helping those with OUD gain understanding—our goal is not to substitute for risk assessment using prediction models [72]. Application of gathered insights from the extracted posts can help decrease stigma and clarify wrong assumptions made about drug misusage and suicidal ideation.

### Conclusions

The goal of this paper is to make use of big data from Reddit to detect suicidality among opioid users. The structure of Reddit offers categories for data sets, and the social media setting can provide case specifics that may not be obtainable elsewhere. This study can serve as a proof of concept for the use of social media site attributes to aid in machine learning methods and for research directions on the feasibility of NNs to abstract textual data and perform in independent areas. A comparison with a high-performing baseline model implies that the absence of hard-coded features may allow more flexibility and accuracy for models when running on out-of-sample data.

## Appendix

Multimedia Appendix 1 Classification metrics using combinations of different model inputs.

Multimedia Appendix 2 Illustration of workflow for C1.

## Abbreviations

API: Application Programming Interface
ATTENTION: attention-based bidirectional recurrent neural network
CNN: convolutional neural network
FAST: FastText
GloVe: Global Vectors for Word Representations
LR: logistic regression
MTURK: Amazon Mechanical Turk
NN: neural network
OUD: opioid use disorder
PRAW: Python Reddit API Wrapper
RF: random forest
RNN: recurrent neural network
SVM: support vector machine
TF-IDF: term frequency-inverse document frequency
