# Supplementary figures and images for: Detection of Suicidality Among Opioid Users on Reddit: Machine Learning–Based Approach
Source: J Med Internet Res. 2020 Nov 27;22(11):e15293. doi: 10.2196/15293 (PMC7732714; doi:10.2196/15293)

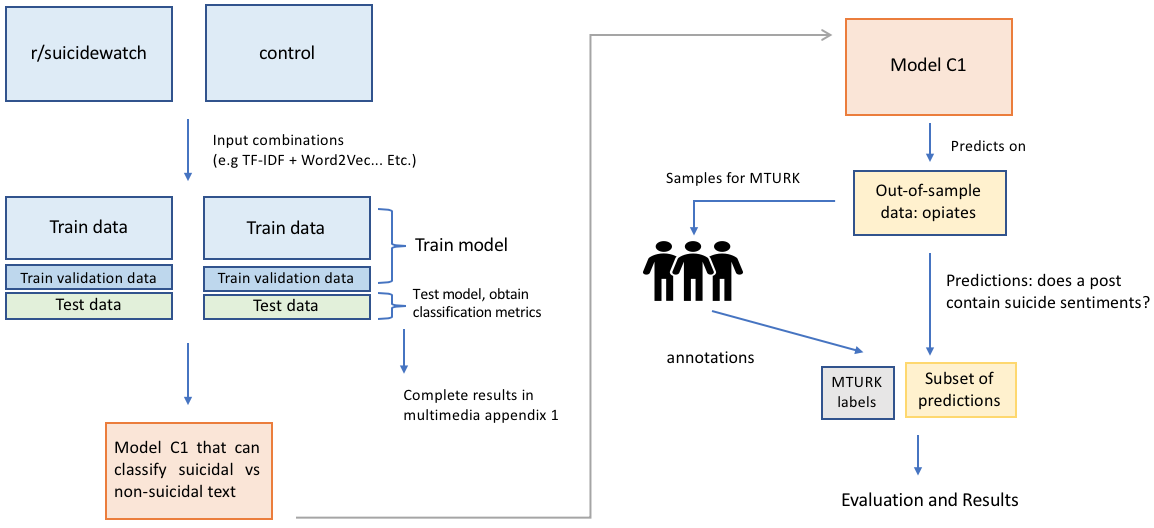

Supplement: Multimedia Appendix 2 [file jmir_v22i11e15293_app2.png]
